# Use of antidepressants and benzodiazepine-related hypnotics before and after initiation of TNF-α inhibitors or non-biological systemic treatment in patients with rheumatoid arthritis, psoriatic arthritis or ankylosing spondylitis

**DOI:** 10.1186/s41927-019-0106-3

**Published:** 2020-02-12

**Authors:** Philip Brenner, Anna Citarella, Louise Wingård, Anders Sundström

**Affiliations:** Department of Medicine Solna, Centre for Pharmacoepidemiology, Karolinska Institutet, SE-171 76 Stockholm, Sweden

**Keywords:** Epidemiology, Antidepressants, Sleeping agents, Rheumatoid arthritis, Psoriatic arthritis, Ankylosing spondylitis, Depression

## Abstract

**Background:**

Rheumatoid arthritis (RA), psoriatic arthritis (PsA) and ankylosing spondylitis (AS) are autoimmune disorders associated with an increased risk for depression, anxiety and sleeping problems. The objective of this study was to analyze use of antidepressants and benzodiazepine-related hypnotics (BRH) in Sweden before and after first time treatment with anti-TNF and non-biological systemic (NBS) treatments among patients with the above diagnoses, and to correlate such use with that of randomly selected population controls.

**Methods:**

Patients and dispensed drugs were identified in nationwide Swedish healthcare registers. Proportions of subjects filling prescriptions of antidepressants and BRH from 2 years before start of treatment (index-date), and 2 years after index date were assessed. Using the period -6 months to index-date as reference, prevalence rate ratios were computed for 6 months’ intervals before and after index. For up to ten randomly selected population controls per patient, the same measures were calculated.

**Results:**

A total of 6256 patients started anti-TNF treatment, and 13,241 NBS treatment. The mean age at index was 52.0 for the anti-TNF group and 56.1 for NBS. Use of antidepressants and BRH was similar in both treatment groups (10.4–12.8%), significantly more common than in the controls (6.6 to 7.6%). For all patients, proportions filling prescriptions for antidepressants and BRH decreased directly or soon after the index; no such changes were seen in the controls, who all showed a slow but steady increase in use over time. Starters of anti-TNF treatment did not show clearer decreases in use of psychotropics than those initiating NBS.

**Conclusions:**

Decreased rates of dispensed psychotropic drugs after the time of anti-TNF and NBS treatment initiation were seen among patients with autoimmune disorders but not population controls. This may correspond to treatment effects of anti-TNF and NBS also on psychiatric symptoms among these patients.

## Introduction

Rheumatoid arthritis (RA), psoriatic arthritis (PsA) and ankylosing spondylitis (AS) are chronic inflammatory systemic diseases primarily affecting the joints [1–3]. This group of autoimmune conditions is associated with a high risk for psychiatric comorbidities, particularly depression, anxiety and sleeping problems [4–6]. RA patients have an estimated point prevalence of depression of 16.8% (95%CI10–23%), which is significantly higher than in the general population [4, 7]. Among patients starting biologic therapy with tumor necrosis factor-α inhibitors (anti-TNF) the proportion with comorbid depression is 19% [8]. Comorbid depression has been associated with poorer response to anti-inflammatory treatment [9, 10], increased work disability [11] and mortality [12] in inflammatory rheumatic disease. The risk for being diagnosed with an anxiety disorder, or mixed anxiety and depression, is elevated for patients with both RA [13] and AS [14], and anxiety may be the symptom with the strongest impact on quality of life among patients with PsA [15]. Sleeping problems are common and among the factors which have the greatest impact of the quality of life among patients with RA [16] and PsA [17], and the risk is elevated among patients with AS as well [14].

There are several theories behind the high rates of psychiatric comorbidity among patients with auto-immune disease. A growing body of evidence indicates that cytokine-mediated communication between the immune system and the brain play a role in the pathogenesis of depression [18–20]. This is further supported by the high prevalence of depression (25%) in hepatitis C patients who have received interferon treatment, a potent inducer of cytokines, and the development of depressive symptoms following experimental immuno-activation in healthy individuals [21, 22].

Conversely, several anti-inflammatory drugs with different mechanisms of action have been studied as treatments in major depression. A review published in 2014 suggested that non-steroidal anti-inflammatory drugs (NSAIDs) are more effective than placebo in treating depression [23]. A recently published meta-analysis of 16 studies on anti-cytokine treatment showed a pooled effect estimate from randomized controlled trials of 0.40 for anti-cytokine treatment vs placebo [24]. Notably, this effect size is comparable to that observed for common antidepressants among patients with major depression [25]. Accordingly, anti-TNF treatment has been associated with lower rates of depression in RA patients [6], and a decreasing proportion of depressed patients over time [26].

It is, however, unclear whether anti-TNF are more effective against psychiatric symptoms than other therapies among patients with autoimmune disease. Rates of anxiety, depression and suicidal ideation in one cross-sectional study were higher among RA patients with anti-TNF compared to other treatments, which the authors suggested would be due to these patients having a higher burden of disease rather than anti-TNF being less effective against psychiatric symptoms [27]. Although there is lack of longitudinal studies on patients with RA, PsA or AS, a recent longitudinal study on patients with skin psoriasis – another immune-mediated condition – showed that patients with anti-TNF had a lower incidence of depressive symptoms than those receiving other therapies, although they had a higher disease burden [28].

Due to a lack of investigations, it is unclear which treatment patients with RA, PsA and AS receive today for psychiatric comorbidities and what treatment patterns look like during different phases of disease. Clinical trials and recommendations are scarce for patients with RA [29] and virtually non-existent among patients with PsA and AS [14, 30, 31]. Observational reports are few, with the exception that Selective Serotonin Reuptake Inhibitors (SSRIs) seem to be around 50% more common among patients with RA compared to other health-care seeking patients [32]. In the general population, SSRIs and other antidepressants is the first-line treatment recommendation for both depression and anxiety in several national treatment guidelines, including in the UK and in Sweden [33–35]. The most commonly prescribed drug class for sleeping problems are benzodiazepine-related hypnotics (BRH, zolpidem or zopiclone, also known as “z-drugs”), although they are recommended only for short-term use due to potential for dose escalation, abuse and dependence [36, 37].

If the evidence summarized above holds true, treatment start with anti-TNF or NBS may alleviate the need for psychotropic drug use in this patient population. We aimed to investigate rates of use of antidepressants and BRH, respectively, relative to time of treatment initiation with anti-TNF or conventional non-biologic systemic (NBS) drugs in a nationwide Swedish population based cohort of patients with RA, AS or PsA compared to the background population. Our hypotheses were that a) rates of antidepressants and BRH would be higher among patients with auto-immune immune disease than in the general population, b) that these rates would be lowered by initiation of disease-modifying treatment, and c) that prescription rates of antidepressants and BRH might be lower after initiation of anti-TNF treatment compared to BRH.

## Methods

### Study design and data sources

This study was a register-based cohort crossover study. Data was obtained from longitudinal population-based health registers maintained by the Swedish National Board of Health and Welfare. Study participants were identified through the National Patient Register (NPR), which contains nation-wide information on all hospitalizations in Sweden since 1987. Outpatient visits in specialized healthcare have been registered since 2001. Dates of hospitalizations and specialist outpatient visits are documented in the register, along with diagnoses assigned by the treating physician. Since 1997 and onwards, diagnoses have been recorded in accordance with the International Classification of Diseases, 10th revision (ICD-10). Information on dispensed drugs was obtained from the Prescribed Drug Register (PDR), which holds information on all prescription fills made in Swedish pharmacies since July 1, 2005, including e.g. date of dispensing, the dispensed quantity, substance name and Anatomical Therapeutic Chemical (ATC) Classification code. Dates of deaths were retrieved from the Cause of Death Register (CDR), and dates of emigration from the Register of the Total Population (maintained by Statistics Sweden); the latter register was also used for identifying population controls. Individual record linkage between the data sources was possible through the unique personal identification number assigned to each Swedish resident.

### Study population

We identified all Swedish residents with a main diagnosis of RA (ICD-10 codes: M05.8, M05.9, M06.0, M06.8 or M06.9), PsA (ICD-10 codes: L40.5, M07.3) or AS (ICD-10 codes: M45, M46.8, M46.9) in the NPR between January 1, 2009, and December 31, 2013. Of these, individuals with at least one filled prescription of anti-TNF or a non-biologic systemic drug between January 1, 2009, and December 31, 2011, and without such fillings during 3 years before the index date, were included in the study. Those treated with these drugs were thus new users in a 3 year time window. The date of the first filling of a study drug between January 1, 2009 and December 31, 2011 was defined as the index date.

Participation further required that the subject was 18 years or older at the time of the first filling and that he or she had been a Swedish resident for at least 3 years, in order to have a 3 year medical history before medication start available in the health registers.

Up to ten population controls were identified to each treated study subject, matched by year of birth, sex and county of residence in the year of the index date of the diseased study subject.

### Exposures

Treatment with anti-TNF and/or non-biologic systemic agents approved for the treatment of RA, PsA and/or AS was our exposure. The studied anti-TNF agents were infliximab (ATC code L04AB02), etanercept (L04AB01), adalimumab (L04AB04), golimumab (L04AB06), and certolizumab (L04AB05). The studied non-biologic systemic agents were methotrexate (ATC-code L01BA01, L04AX03), sulfasalazine (A07EC01), betamethasone (H02AB01), methylprednisolone (H02AB04), prednisolone (H02AB06), prednisone (H02AB07), triamcinolone (H02AB08), and leflunomide (L04AA13).

The duration of treatment was estimated on the group level; treatment was considered as continuous as long as the interval between fillings of anti-TNF or NBS was less than 180 days.

### Observation time and outcomes

The outcome measures were proportions of study subjects with filled prescriptions of antidepressants (ATC-code N06A) and benzodiazepine related hypnotics (ATC-code N05CF) during the observation period. While diagnostic data in the NPR only covers specialized health care, using prescription data from the PDR allowed us to identify patients treated for psychiatric symptoms also on the primary care level. Observation started 2 years before the index date and ended at the completion of 2 years of treatment. Study subjects were censored at end of treatment, end of follow-up or switch of treatment (from anti-TNF to non-biologic systemics; or from non-biologic systemics to anti-TNF). Addition of a NBS to anti-TNF regimen was not a cause for censoring. Finally, censoring was also made at emigration or death.

In order to describe general morbidity and health care utilization before index date, the following variables were assessed in the study population: health care resources as measured by number of out-patient specialist visits, the proportion of the population with at least one hospitalization during the year preceding the index date, and the number of prescribed drugs (unique ATC codes) during 3 months before index. This is because a prescription in Sweden generally is for 3 months’ supply (100 pills).

#### Main outcomes

The proportion of study subjects that filled a prescription of either of the outcome drugs was estimated in 6 month intervals 2 years before and after the index date. The following periods were defined: between 24 and 18 months, 18 and 12 months, 12 and 6 months, and the 6 months before the index date. This latter period constituted the reference period in the cohort crossover analysis. After index, the following periods were identified: the first 6 months after, between 6 and 12 months, 12 and 18 months and 18 to 24 months after index date.

**Fig. 1:**
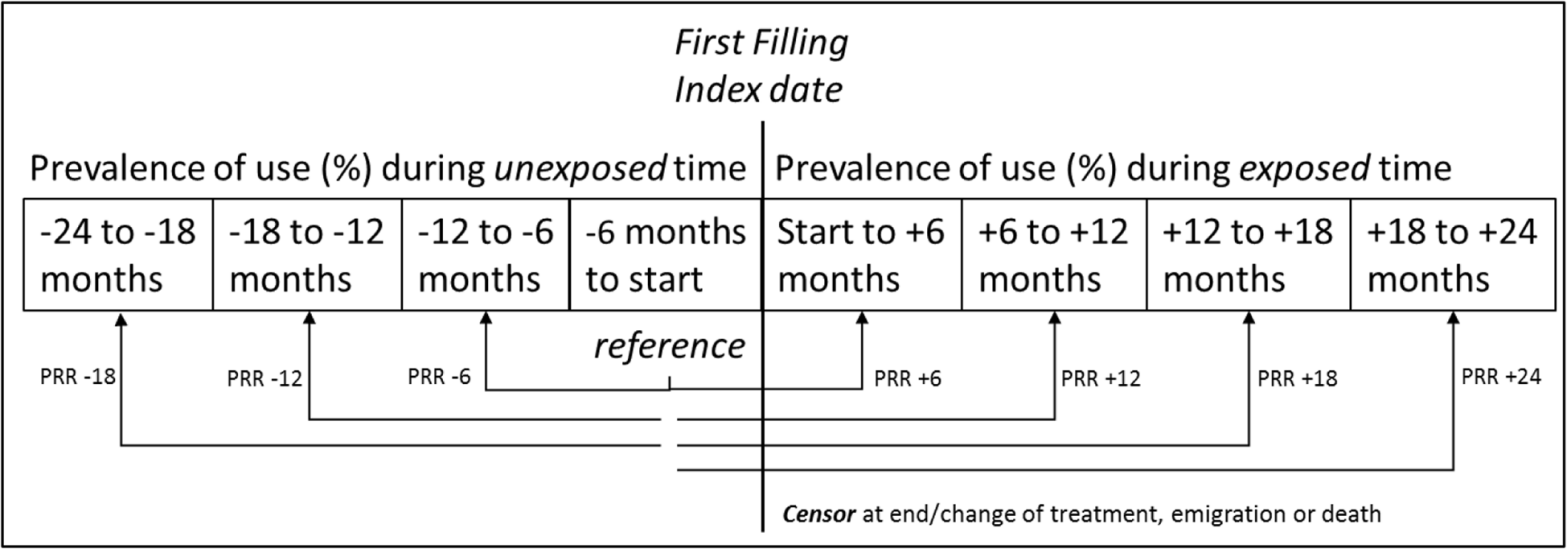
Periods of observation time

The nominator was the number of individuals who had filled at least one prescription during the period. Any individual could fill prescriptions during more than period and therefore be counted in the nominator in several periods. The denominator was the total number of individuals eligible for prescriptions at start of each 6 months’ interval. Periods of observation time are illustrated in Fig. 1.

Prevalence rates of users per 100 study subjects per 6 month interval were calculated, and using the period closest to the index-date, i.e. the 6 months before start of either anti-TNF or NBS, as reference rate, prevalence rate ratios (PRR) (with 95% confidence intervals, CI) were calculated for the intervals before and after the reference period. PRRs were calculated for the three intervals before the index period as well as for the four intervals after the index period.

Furthermore, the patterns of use of the outcome drugs were also assessed in the background population by calculating PRRs per time intervals among the randomly selected population controls, matched by year of birth, sex and county of residence at the year of the index date, in a ratio of up to 10 controls per diseased study subject. Changes in use over time could therefore be contrasted to those in the diseased and treated study subjects.

Controls were excluded if they had a diagnosis of RA, AS or PsA before the index-date of the matched diseased study subject; and they were censored from follow-up at emigration, death or a recorded diagnosis of RA, AS or PsA.

Finally, sensitivity analyses were performed in the population starting anti-TNFs: patterns of use before and after start of the separate antibodies infliximab, etanercept, adalimumab, certolizumab pegol and golimumab were assessed. Sensitivity analyses were also performed for each separate diagnosis group (RA, AS or PsA).

## Results

Characteristics of the patient cohorts and the population controls are shown in Table 1. We identified 3697 RA patients with TNF initiation and 7996 patients with NBS initiation. Corresponding numbers for PSA were 1223 and 3275 patients, and for AS 1336 and 1970 patients. The proportion of women was higher than that of men among RA patients in both the anti-TNF and NBS-treated groups (74 and 69% women), similar among PsA patients (50% in both treatment groups) and lower among AS patients (37 and 43%). The mean age of patients starting anti-TNF treatment was lower than those receiving NBS regimens (RA: 56 vs 60 years: PSA: 49 vs 51 years; AS 42 vs 47 years). Table 1 also shows the baseline (the period 6 months before index date) rate of use of the outcome drug groups antidepressants and BRH.

**Table 1:** Characteristics of patients and controls

	TNF		NBS	
N	6256		13,241	
Women, %	61.6		60.5	
Age mean (SD)	52.0	(14.5)	56.1	(16.3)
*Women*	*53.1*	*(14.5)*	*56.0*	*(16.6)*
*Men*	*50.3*	*(14.3)*	*56.3*	*(15.8)*
Antidepressants				
Baseline rate, %	11.8		10.9	
Controls, %	6.6		7.6	
Difference (95% c.i.)	5.2	(4.35–5.99)	3.3	(2.77–3.87)
Benzodiazepine-related hypnotics				
Baseline rate, %	10.7		10.4	
Controls, %	6.6		7.6	
Difference (95% c.i.)	4.1	3.34–4.92	2.8	2.25–3.33
Number of drugs (substances, 3 months before index)				
Patients				
mean (SD)	6.9	(4.2)	6.5	(3.8)
Controls				
mean (SD)	2.0	(3.0)	2.3	(3.1)
Number of outpatient visits (past 12 months)				
Patients				
mean (SD)	5.6	(4.1)	3.0	(3.7)
Controls				
mean (SD)	0.8	(2.3)	0.8	(2.4)
Proportion, at least one hospitalization (past 12 months)				
Patients, %	20		21	
Controls, %	9		10	

*TNF* TNF-α inhibitors, *NBS* Non-biologic systemic

**Fig. 2:**
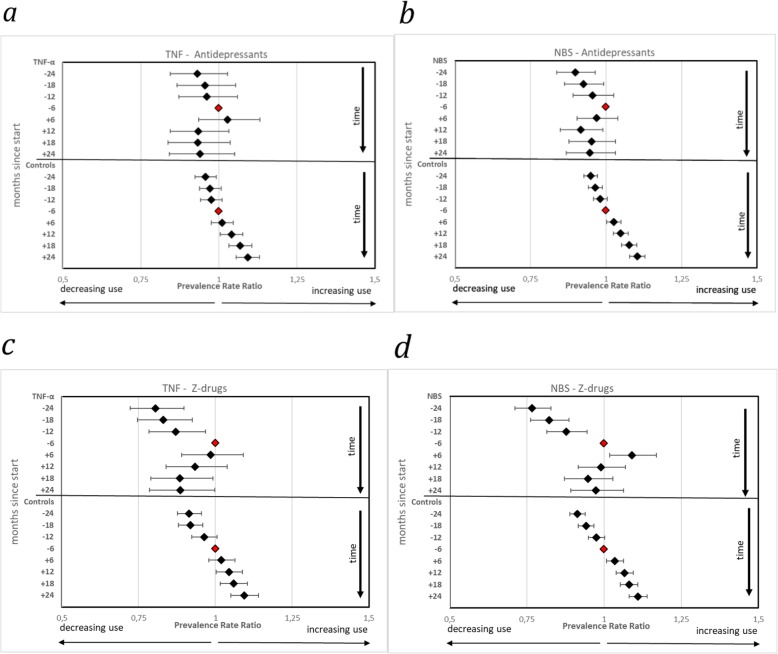
**a–d** Prevalence Rate Ratios of individuals filling prescriptions of antidepressants and benzodiazepine related hypnotics (Z-drugs) in patients with inflammatory disorders and their matched controls before and after treatment initiation with TNF-α inhibitors (TNF) and non-biologic system agents (NBS). Reference is the period minus 6 months to index date (marker without confidence interval in graph)

The results from the analysis on changes in the proportion of patients and controls filling a prescription of *antidepressants* before and after start with anti-TNF – measured by prevalence rate ratios (PRR), using the period minus 6 months to index date as reference - is shown in Fig. 2a, and for NBS treatment in Fig. 2b. While the use among population controls increased slightly but steadily over the time period, both patient groups showed marked decreases in antidepressant use after initiation of anti-TNF and NBS. CIs for all time periods were, however, overlapping with those of the index period, except for the CI of the NBS group, period + 6–12 months.

In Fig. 2c and d, the results for filled prescriptions of *BRH* are shown. As for ADs, the PRR in both control populations display a steady increase during observation, however with an interruption during the actual reference period in NBS controls. The treated populations also show similar patterns as for antidepressants: in the anti-TNF population, the decrease in use is seen immediately after the index, and statistically significantly PRRs are observed after 12 months of follow-up. As regards the NBS population, the PRR actually show a significant increase also in the first 6 months after the index date, but thereafter the increase in use is halted.

Population size and absolute number of users per time interval and per indication group are presented in Additional file 1: Tables S1–S9.

**Fig. 3:**
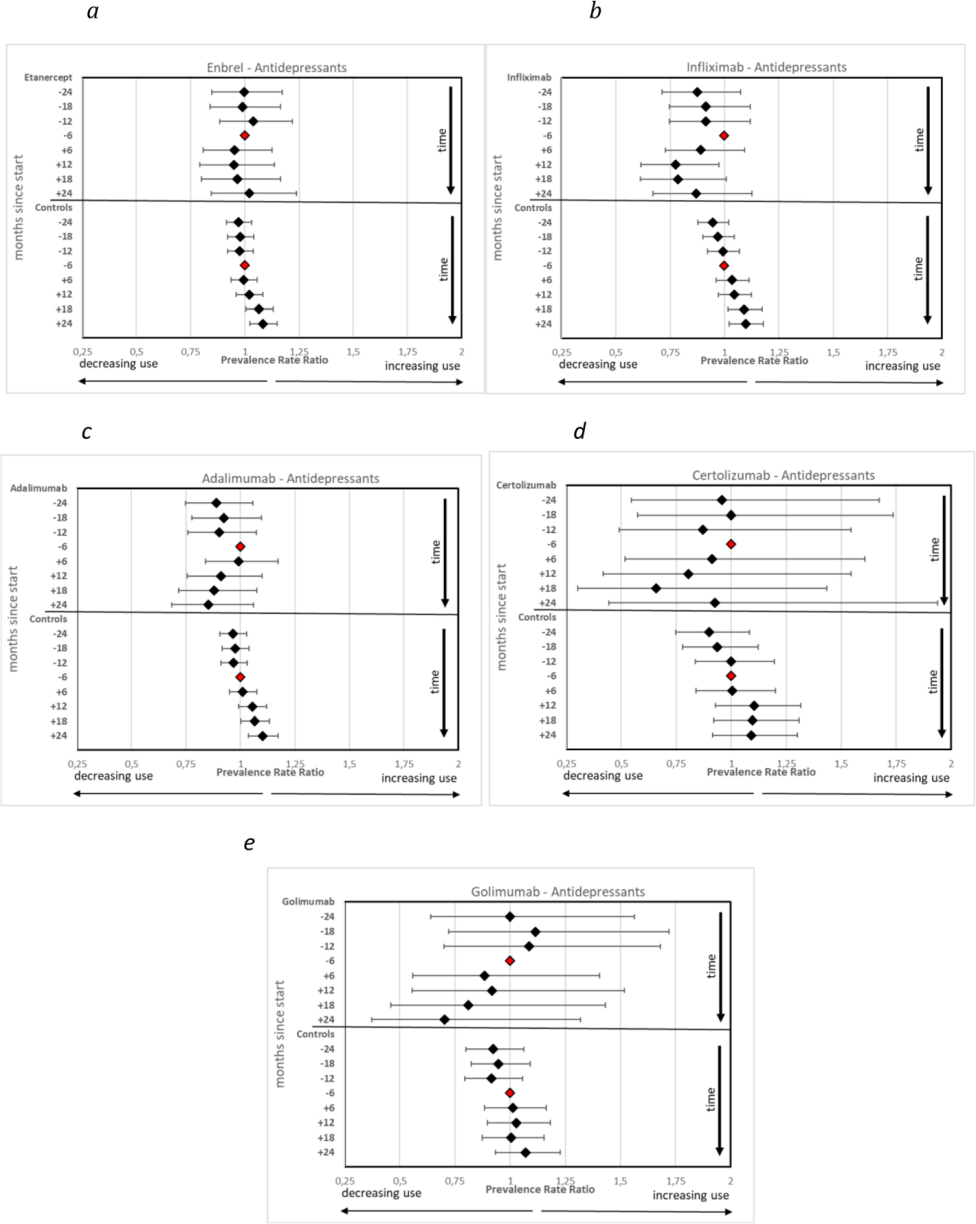
**a-e** Prevalence Rate Ratios of individuals filling prescriptions of antidepressants in patients with inflammatory disorders and their matched controls before and after treatment initiation with TNF-α inhibitors (TNF), per specific drug. Reference is the period minus 6 months to index date (marker without confidence interval in graph)

**Fig. 4:**
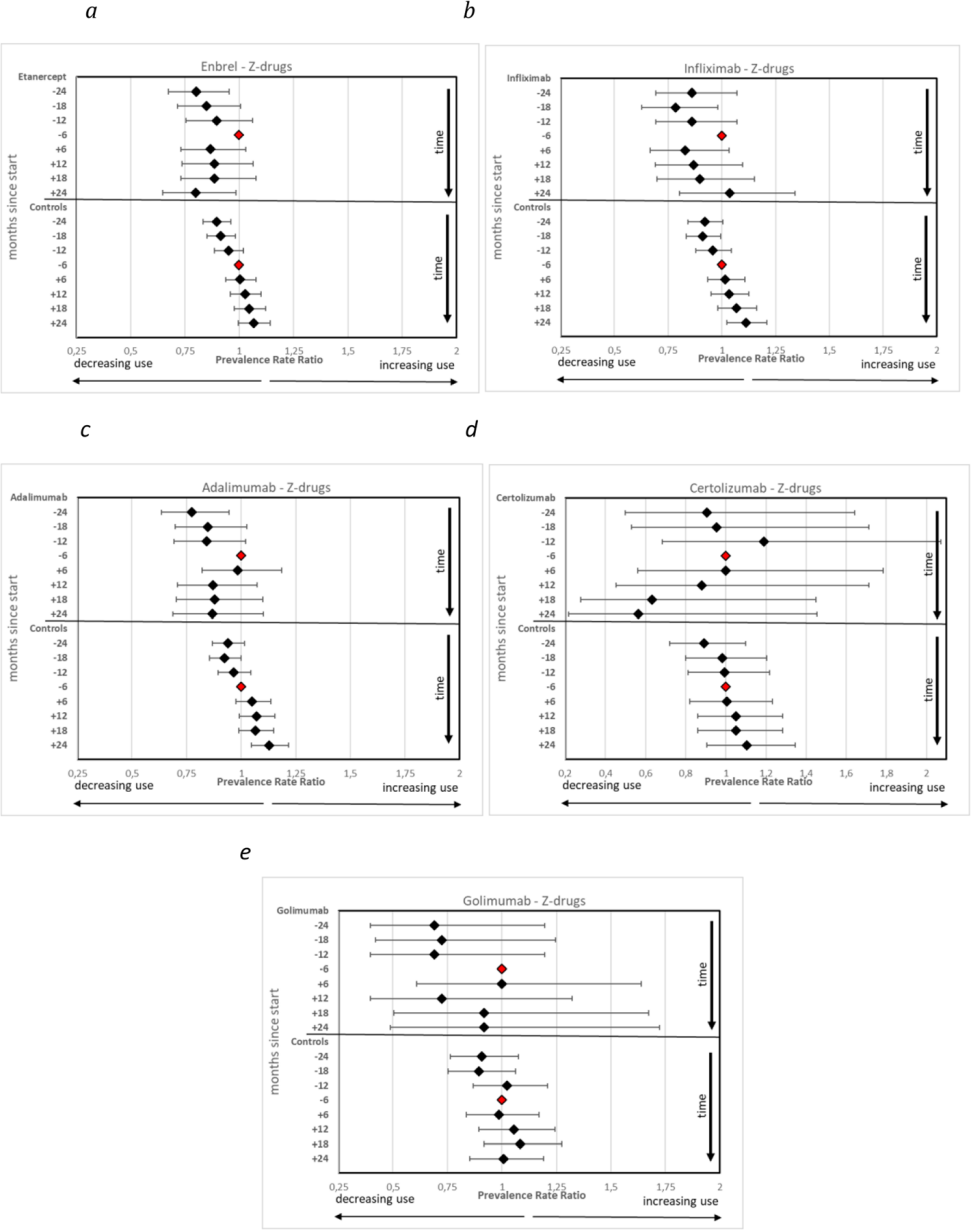
**a**-**e** Prevalence Rate Ratios of individuals filling prescriptions of benzodiazepine related hypnotics (Z-drugs) in patients with inflammatory disorders and their matched controls before and after treatment initiation with TNF-α inhibitors (TNF), per specific drug. Reference is the period minus 6 months to index date (marker without confidence interval in graph)

Results of the sensitivity analyses illustrating patterns of use of antidepressants and BRH before and after start of specific anti-TNF substances are showed in Fig. 3a–e (use of antidepressants) and Fig. 4a-e (use of BRH). Similar patterns are seen for both treated populations and controls: marked changes in use after index-date for treated populations, and steady increases in use for controls; the confidence intervals are however very wide for certolizumab and golimumab.

It can also be noted that the decrease of use of antidepressants after start of etanercept is much smaller than the decrease after start of both infliximab and adalimumab.

In the sensitivity analyses separating the three diagnostic groups (Additional file 1: Figures S1a–f and S2a–f), patterns were similar; however, number of patients were small in the PsA and AS groups and CIs wide.

## Discussion

This is the first study to evaluate the use of antidepressants and BRH in a cohort of patients with inflammatory disorders before and after treatment start with anti-TNF or NBS. Our results show that before treatment start, rates of both psychotropic substance groups increased among the patients - similar to the matched population control subjects. In contrast, after start of anti-TNF or NBS, the rates of psychotropic drugs diminished or levelled out, while the increase among population controls continued.

The increasing rates of psychotropic drugs in the patients groups before treatment start may correspond to an increase in psychiatric symptom burden due to inadequate inflammatory disease symptom management [38]. This explanation is consistent with several studies that demonstrate the association between psychiatric symptoms and greater pain, poorer functional status and worse arthritis disease outcomes, especially in patients with early diagnosis and before treatment start [39–44].

A possible mechanism behind this association is that the inflammatory component of the investigated disorders may play a direct role in the increase of psychiatric symptoms. A growing body of evidence supports the role of inflammation in the development of psychological distress, especially depression [20, 45–48]. A meta-analysis of 82 studies provided evidence that peripheral levels of several pro-inflammatory cytokines (i.e. interleukin-6 and TNF-α) were more elevated in patients with major depression disorder compared to healthy controls [49]. Furthermore, about 25% of patients with chronic hepatitis C develop depression after treatment with the pro-inflammatory cytokine interferon-α [50]. Immune-inflammatory pathways dysregulation and cell-mediated immunity activation may play an important role in inducing functional and structural brain changes involved in the pathophysiology of depression [18].

Additionally, a psychological reaction may be associated with the prospective of living with a chronic disorder, the uncertainty of future impact on physical disability and psychosocial consequences, or may be due to disease symptoms (i.e. pain or insomnia) [51]. Both cross-sectional and longitudinal studies have shown the complex relation between the disease activity and severity of comorbid psychiatric disorders [38]. In our diseased population, the increasing use of BRH may be also attributable to symptomatic treatment of sleep problems directly caused by pain and joint stiffness [52].

After the start of treatment with TNF-α or NBS medications, rates of psychotropic drugs tended to level out or decrease in the diseased population, although differences in drug rates between the index period and the subsequent time periods were not always statistically significant. This pattern differed from that of the population controls. In general, patients with anti-TNF had higher rates of psychotropics before treatment start than did patients treated with NBS although they were in general younger, corresponding to the assumption that patients eligible for the latter treatment have a higher disease burden.

These decreasing rates of psychotropic drugs could be interpreted as a lessened need for treatment of depression and sleeping problems among the treated patients caused by a direct and/or indirect treatment effect. Uguz and colleagues showed that psychiatric disorders were identified less frequently in patients with RA receiving anti-TNF compared to patients who did not receive such medications [6], and a significant decrease in depression has been reported in patients with AS who receive anti-TNF [53].

Despite the different mechanism of action of anti-TNF compared to NBS, no clear difference could be seen regarding rates of psychotropic drugs after treatment initiation. The study design did not allow for meaningful comparison regarding any difference between the two treatment groups as the patients showed differing characteristics at baseline regarding both overall morbidity and psychotropic drug rates. In separate analyses regarding both individual anti-TNF compounds and the three different diagnoses, patterns were similar but with wider CIs. Nevertheless, the reduced rates of dispensed psychotropic medications, especially observed after 6–12 months for both treatment groups, may correspond in time with established and maintained effect on disease symptoms [54]. An effective treatment of rheumatic disease symptoms could lead to an improvement in disease activity and pain that in turn could be associated with an improvement on mood and sleep, although this mediating role may be hard to disentangle tangle from direct effects of the treatment on psychiatric symptoms [24]. Given the established link between psychiatric symptomatology and immunomodulatory mechanisms, this may also be due to a direct effect of the treatment on pro-inflammatory mechanisms [18–20].

Patients treated with anti-TNF drugs have a slightly increased risk for psychiatric side effects such as depression, anxiety and insomnia that may increase the rate of psychotropic drug use [55]. However, our results seem to support the notion that anti-TNF drugs do not have significant adverse effects for the majority of patients and the psychiatric benefits of these treatments may outweigh the risks [26].

### Strengths and limitations

The main strengths of the present study are the use of data from several nationwide registers as well as the use of a large nationwide cohort [56]. The Swedish registers are validated data sources with highly complete information that have proved to be reliable in many studies [56]. The use of filled prescriptions as a measure of drug exposure is a further strength of our study, as dispensed prescriptions as measure of the outcome eliminates recall bias and improves the accuracy of the information on drug use, and allows for identification of given healthcare on the primary care level, i.e. before patients with inflammatory disorders may have seen a rheumatologist [57].

There are also limitations that need to be addressed. First, this crossover study used self-matching, i.e. comparison within each case, which eliminates confounding by stable and slow-varying characteristics, including unmeasured confounding. However, confounding by characteristics that change over time, such as comorbidity or use of other drugs, is still possible. Secondly, the method of identifying RA, PsA or AS patients through the NPR means that patients who have not received any specialist care during the five-year interval studied were misclassified. Third, lack of information in the PDR regarding indications for prescribing is a limitation. Drugs like antidepressants could have been prescribed not only for anxiety or depression but also off-label for other conditions (e.g. premenstrual syndrome) [58]. Fourth, patients with rheumatic disorders are likely to undergo outpatient visits, which increase the probability that psychological distress is diagnosed and treated compared to non-diseased controls (i.e. surveillance bias).

Lastly, as we did not have access to direct clinical information in this study it remains difficult to distinguish whether the overall reduction in dispensed prescription rates in the diseased group is the result of the direct anti-inflammatory effect of the medications, an indirect effect of the improved rheumatic disease leading to better psychological status, or a combination of both. Further research involving participants with inflammatory disorders and concurrent psychiatric symptoms should assess this interesting question.

## Conclusions

Patients with inflammatory disorders had decreasing prescription rates of antidepressants and benzodiazepine-related hypnotics after initiation of anti-TNF- or NBS treatment. Mechanisms underlying this association should be further investigated.

## Supplementary information

**Additional file 1: Figure S1a–S1 f**. Use of antidepressants among patients with rheumatoid arthritis (RA), psoriatic arthritis (PsA) and ankylosing spondylitis (AS) and their matched controls before and after treatment initiation with TNF-α inhibitors (TNF) and non-biologic systemic agents (NBS). **Figure S2a–S2 f**. Use of zolpidem/zopiclone (Z-drugs) among patients with rheumatoid arthritis (RA), psoriatic arthritis (PsA) and ankylosing spondylitis (AS) and their matched controls before and after treatment initiation with TNF-α inhibitors (TNF) and non-biologic system agents (NBS). **Table S1**. Characteristics of patients and controls by indication for treatment. **Table S1b.** Treatments (TNF-substance) in patients by indication for treatment. **Table S1c**. Treatments (NBS) in patients by indication for treatment. **Table S2**. Patients starting Anti-TNF treatment, proportion with fillings of antidepressants before and after treatment start. **Table S3**. Controls to patients starting Anti-TNF treatment, proportion with fillings of antidepressants before and after treatment start. **Table S4**. Patients starting Anti-TNF treatment, proportion with fillings of benzodiazepine related hypnotics before and after treatment start. **Table S5**. Controls to patients starting Anti-TNF treatment, proportion with fillings of benzodiazepine related hypnotics before and after treatment start. **Table S6**. Patients starting non-biological systemics, proportion with fillings of antidepressants (ADs) before and after treatment start. **Table S7**. Controls to patients starting non-biological systemics, proportion with fillings of antidepressants before and after treatment start. **Table S8**. Patients starting non-biologic systemics, proportion with fillings of benzodiazepine related hypnotics (BRH) before and after treatment start. **Table S9**. Controls to patients starting non-biological systemics, proportion with fillings of benzodiazepine related hypnotics (BRH) before and after treatment start.

## Data Availability

The Ethical Review Board approval for this study was obtained for public sharing and presentation of data on group level only. This means that the data used for this study may not be shared by the authors. Data sources are listed in the Methods section and may be available by any researcher by application to the Swedish governmental agencies National Board of Health and Welfare, and Statistics Sweden.

## Abbreviations

AS: Ankylosing Spondylitis
ATC: Anatomical Therapeutic Chemical (ATC) Classification code
BRH: Benzodiazepine Related Hypnotics
CDR: Cause of Death Register
CI: Confidence Intervals
ICD-10: International Classification of Diseases, 10th revision
NBS: Non-Biologic Systemic
NPR: National Patient Register
PDR: Prescribed Drug Register
PRR: Prevalence Rate Ratios
PsA: Psoriatic Arthritis
RA: Rheumatoid Arthritis
TNF: Tumor Necrosis Factor
