# Supplementary material for: Use of antidepressants and benzodiazepine-related hypnotics before and after initiation of TNF-α inhibitors or non-biological systemic treatment in patients with rheumatoid arthritis, psoriatic arthritis or ankylosing spondylitis
Source: BMC Rheumatol. 2020 Feb 12;4:9. doi: 10.1186/s41927-019-0106-3 (PMC7014636; doi:10.1186/s41927-019-0106-3)
Supplement: Supplementary file 1 — Additional file 1: Figure S1a–S1 f. Use of antidepressants among patients with rheumatoid arthritis (RA), psoriatic arthritis (PsA) and ankylosing spondylitis (AS) and their matched controls before and after treatment initiation with TNF-α inhibitors (TNF) and non-biologic systemic agents (NBS). Figure S2a–S2 f. Use of zolpidem/zopiclone (Z-drugs) among patients with rheumatoid arthritis (RA), psoriatic arthritis (PsA) and ankylosing spondylitis (AS) and their matched controls before and after treatment initiation with TNF-α inhibitors (TNF) and non-biologic system agents (NBS). Table S1. Characteristics of patients and controls by indication for treatment. Table S1b. Treatments (TNF-substance) in patients by indication for treatment. Table S1c. Treatments (NBS) in patients by indication for treatment. Table S2. Patients starting Anti-TNF treatment, proportion with fillings of antidepressants before and after treatment start. Table S3. Controls to patients starting Anti-TNF treatment, proportion with fillings of antidepressants before and after treatment start. Table S4. Patients starting Anti-TNF treatment, proportion with fillings of benzodiazepine related hypnotics before and after treatment start. Table S5. Controls to patients starting Anti-TNF treatment, proportion with fillings of benzodiazepine related hypnotics before and after treatment start. Table S6. Patients starting non-biological systemics, proportion with fillings of antidepressants (ADs) before and after treatment start. Table S7. Controls to patients starting non-biological systemics, proportion with fillings of antidepressants before and after treatment start. Table S8. Patients starting non-biologic systemics, proportion with fillings of benzodiazepine related hypnotics (BRH) before and after treatment start. Table S9. Controls to patients starting non-biological systemics, proportion with fillings of benzodiazepine related hypnotics (BRH) before and after treatment start. [file 41927_2019_106_MOESM1_ESM.docx]

Additional file 1

Figures S1a-S1f. Use of antidepressants among patients with rheumatoid arthritis (RA), psoriatic arthritis (PsA) and ankylosing spondylitis (AS) and their matched controls before and after treatment initiation with TNF-α inhibitors (TNF) and non-biologic systemic agents (NBS).

*Figure S1a Figure S1b*


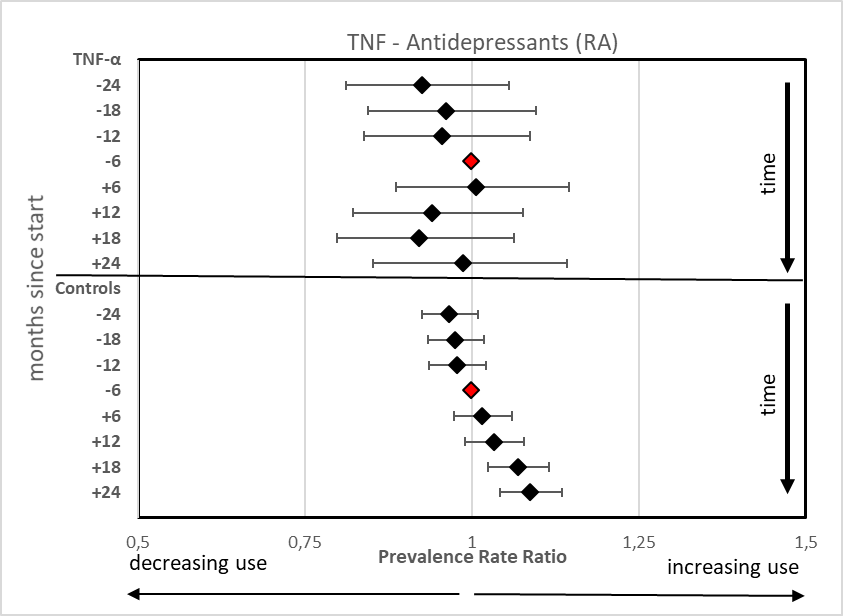

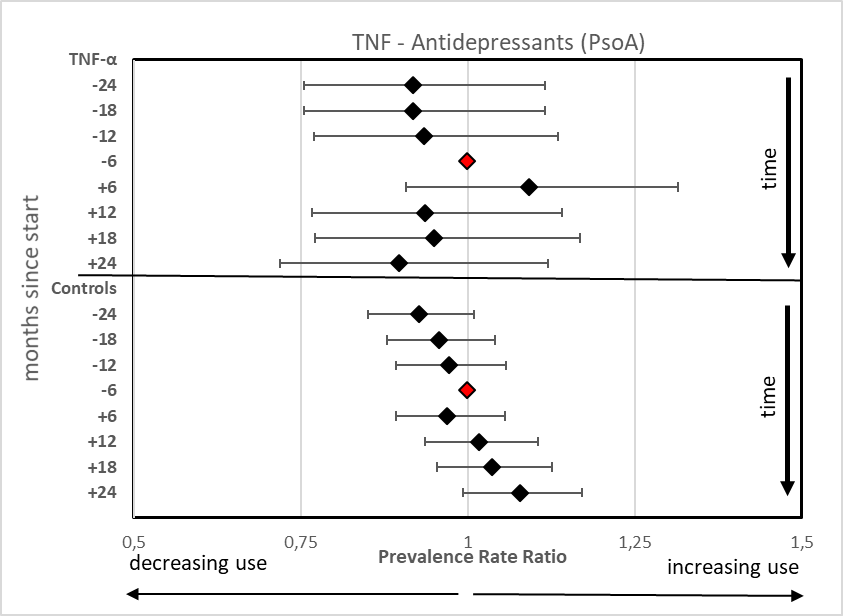


*Figure S1c Figure S1d*


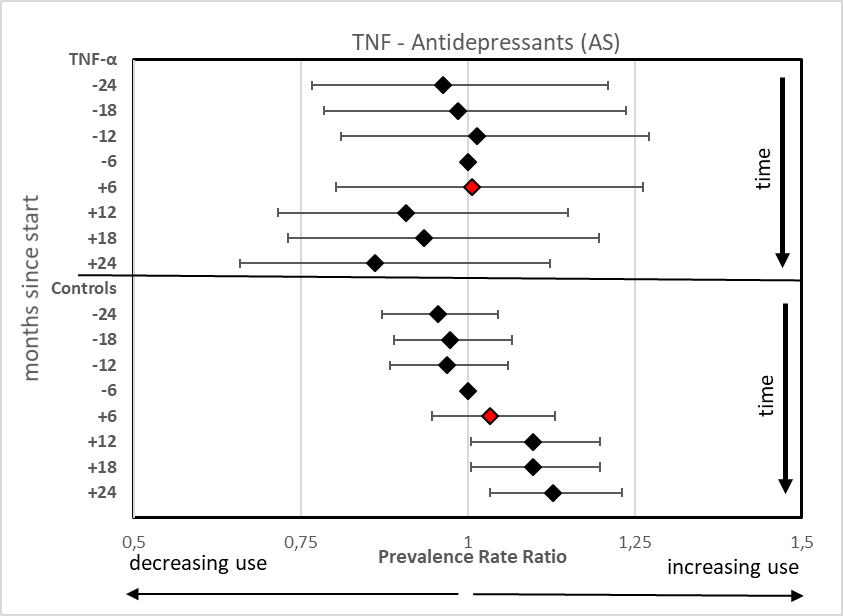

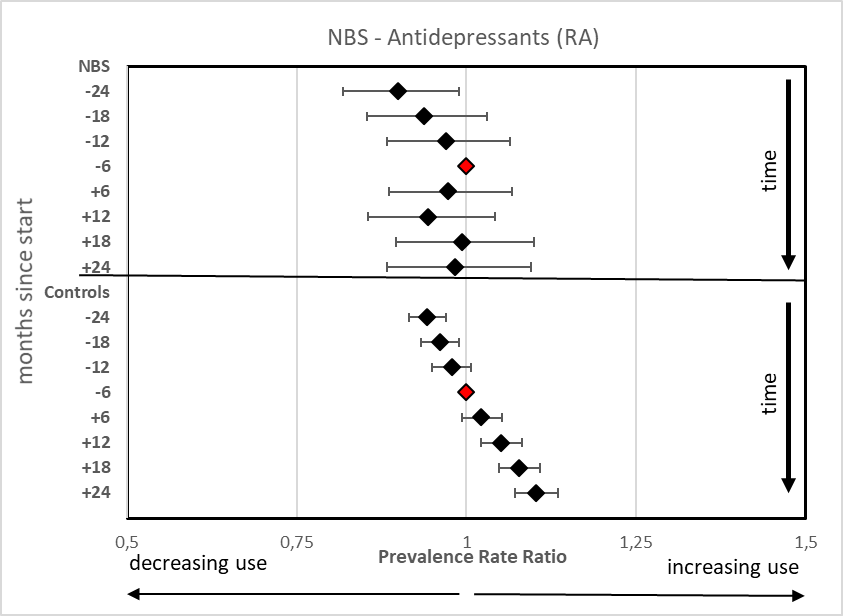


*Figure S1e Figure S1f*


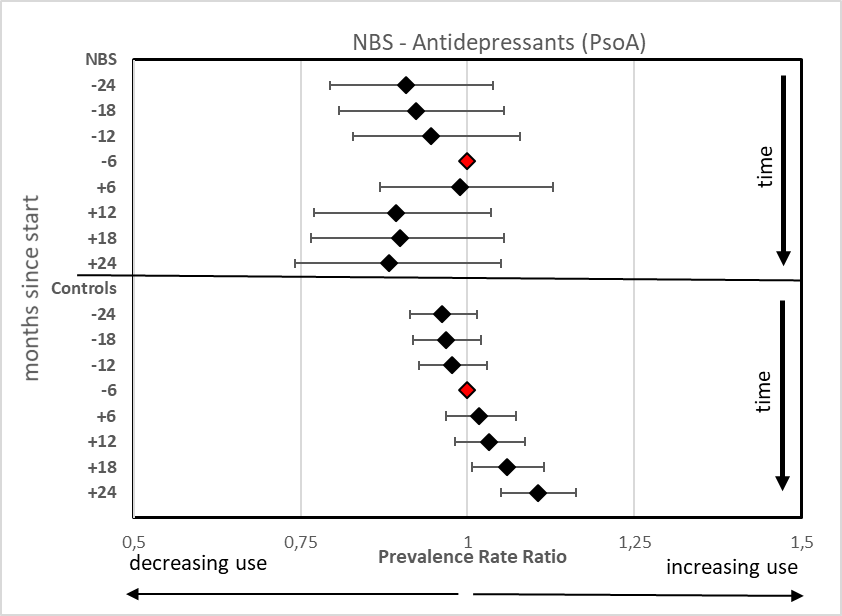

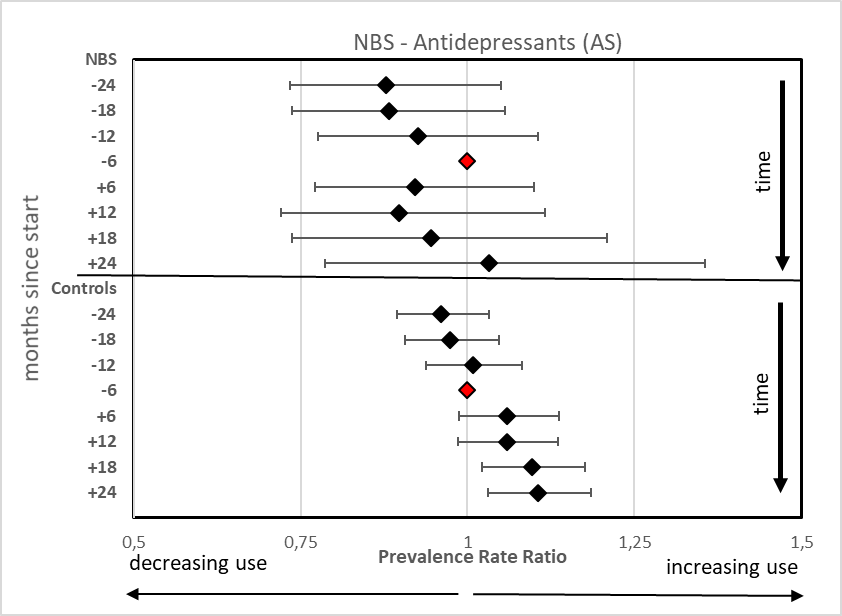


Figures S2a-S2f. Use of zolpidem/zopiclone (Z-drugs) among patients with rheumatoid arthritis (RA), psoriatic arthritis (PsA) and ankylosing spondylitis (AS) and their matched controls before and after treatment initiation with TNF-α inhibitors (TNF) and non-biologic system agents (NBS).

*Figure S2a Figure S2b*


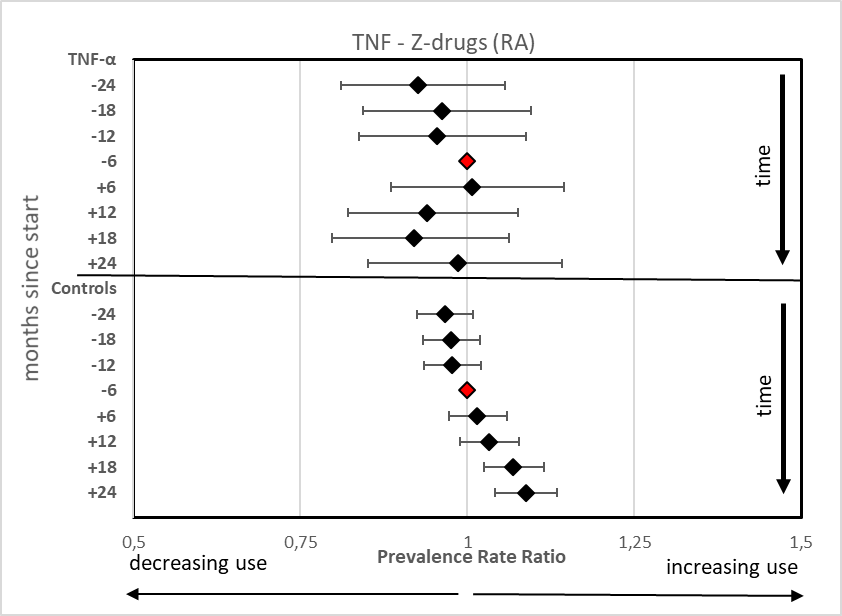

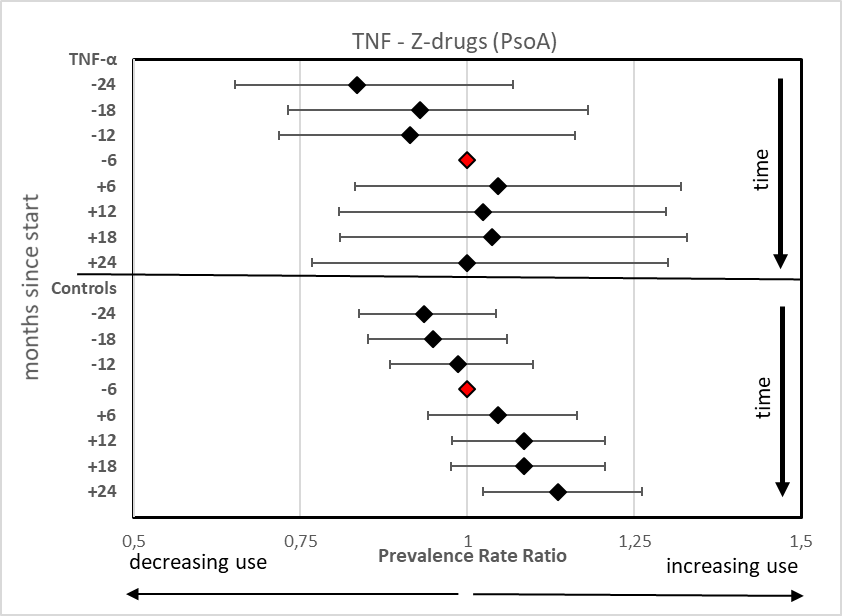


*Figure S2c Figure S2d*


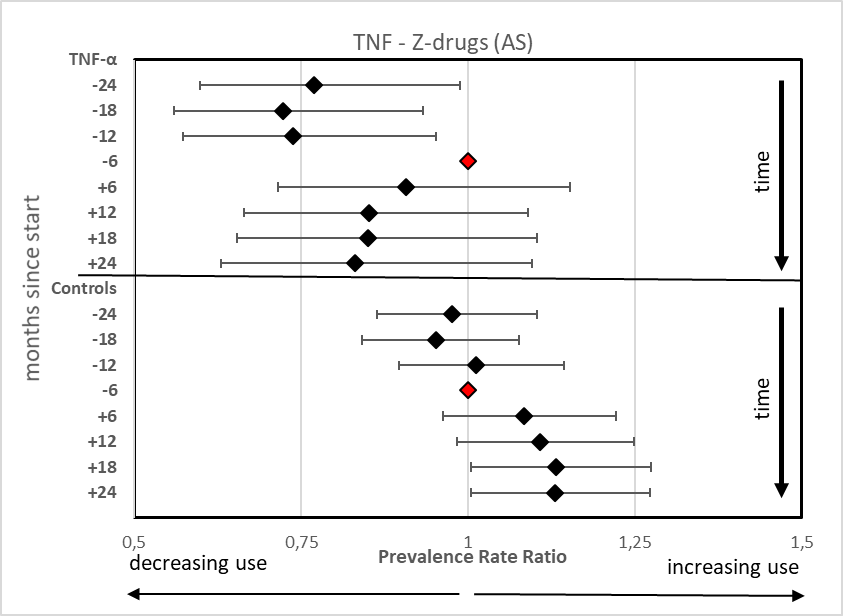

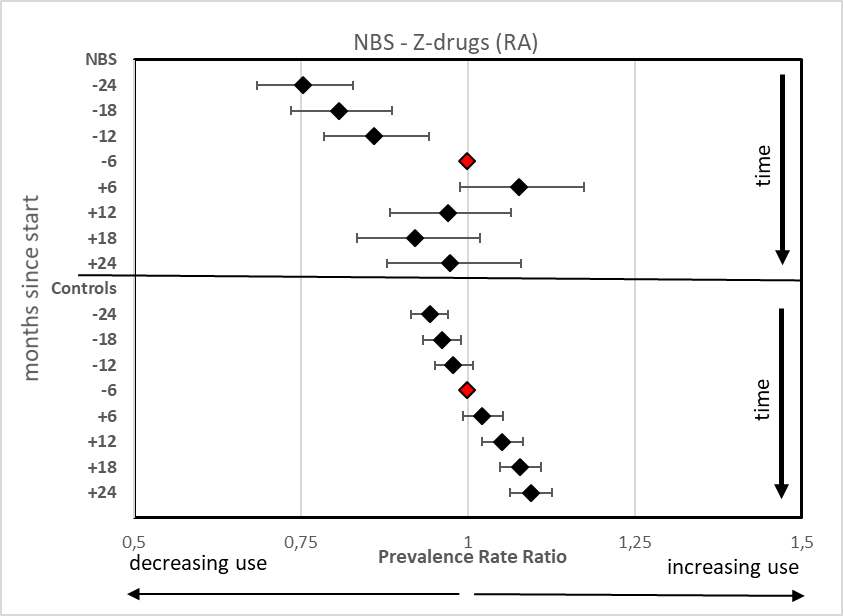


*Figure S2e Figure S2f*


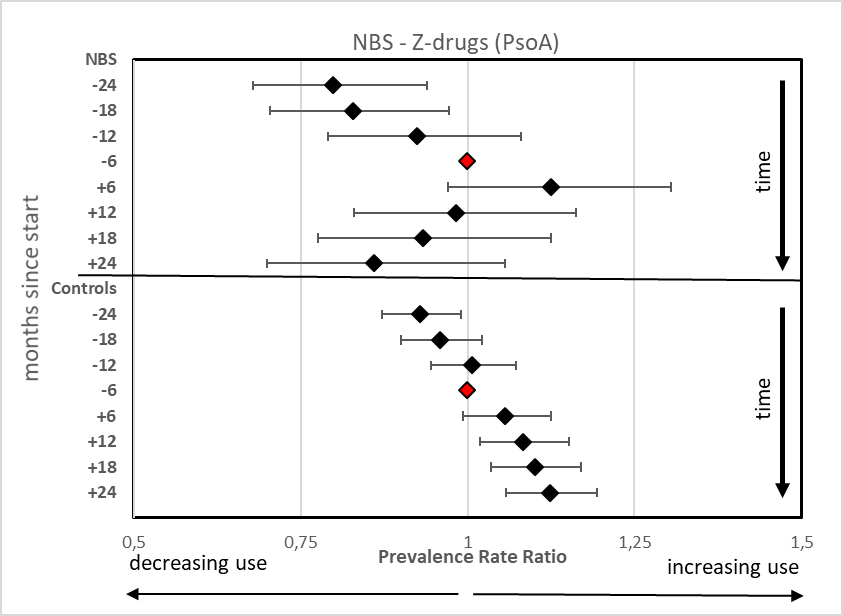

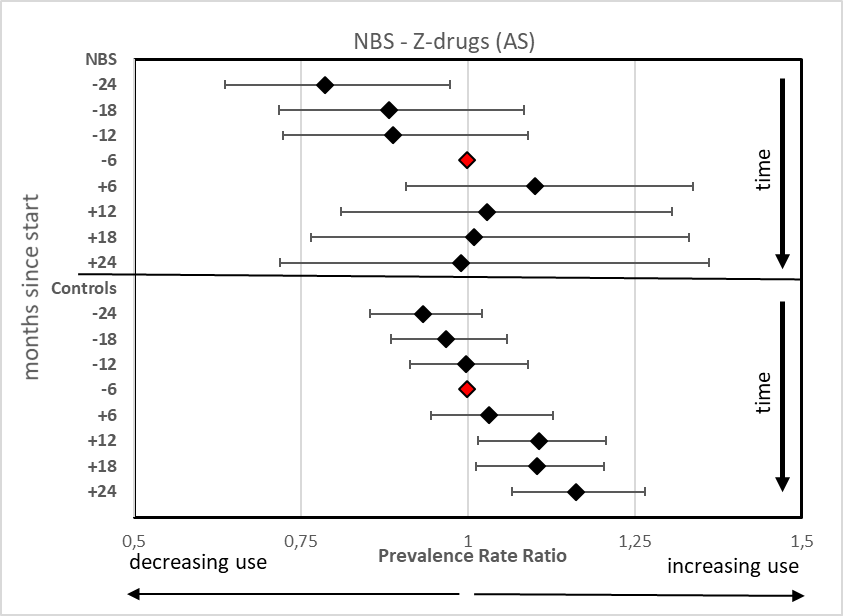


Table S1. Characteristics of patients and controls by indication for treatment

|  | **RA** | | | |  | **PSA** | | | |  | **AS** | | | |
| --- | --- | --- | --- | --- | --- | --- | --- | --- | --- | --- | --- | --- | --- | --- |
|  | TNF |  | NBS |  |  | TNF |  | NBS |  |  | TNF |  | NBS |  |
| N | 3,697 |  | 7,996 |  |  | 1,223 |  | 3,275 |  |  | 1,336 |  | 1,97 |  |
| Females, % | 74.2 |  | 69.2 |  |  | 50.3 |  | 49.7 |  |  | 36.9 |  | 43.4 |  |
|  |  |  |  |  |  |  |  |  |  |  |  |  |  |  |
| age mean (SD) | **56.3** | (14.0) | **60.1** | (15.8) |  | **49.1** | (12.4) | **51.4** | (14.3) |  | **42.9** | (12.6) | **47.4** |  |
| *females* | *55.5* | *(14.4)* | *59.0* | *(16.4)* |  | *50.1* | *(12.5)* | *51.4* | *(14.5)* |  | *43.1* | *(12.2)* | *45.0* |  |
| *males* | *58.3* | *(12.5)* | *62.7* | *(14.1)* |  | *48.2* | *(12.3)* | *51.4* | *(14.1)* |  | *42.8* | *(12.8)* | *49.2* |  |
|  |  |  |  |  |  |  |  |  |  |  |  |  |  |  |
| Disease duration (years) mean (SD) | 8.7 | (9.5) | 1.2 | (3.2) |  | 7.6 | (7.9) | 1.8 | (3.6) |  | 10.1 | (10.7) | 3.2 | (5.6) |
| *Median (1^st^ – 3^rd^ quartiles)* | 5.9 | (2.1-10.8) | 0 | (0 – 0.3) |  | 5.4 | (2.0-9.9) | 0.1 | (0-2) |  | 6.8 | (1.7-15) | 0.5 | (0-5.1) |
|  |  |  |  |  |  |  |  |  |  |  |  |  |  |  |
| **Antidepressants** |  |  |  |  |  |  |  |  |  |  |  |  |  |  |
| Baseline rate, % | 11.3 |  | 10.2 |  |  | 15.0 |  | 12.3 |  |  | 10.2 |  | 11.7 |  |
| Controls, % | 10.1 |  | 10.3 |  |  | 8.3 |  | 8.2 |  |  | 6.7 |  | 7.1 |  |
| Difference (95% c.i.) | 1.2 | (0.78-2.19) | -0.1 | ((-0.84) - 5.53) |  | 6.7 | (4.64-8.76) | 4.1 | (2.97-5.29) |  | 3.5 | (1.80-5.16) | 4.5 | (3.07-6.00) |
|  |  |  |  |  |  |  |  |  |  |  |  |  |  |  |
| **Benzodiazepine related hypnotics** |  |  |  |  |  |  |  |  |  |  |  |  |  |  |
| Baseline rate, % | 11.2 |  | 11.2 |  |  | 10.4 |  | 9.2 |  |  | 9.7 |  | 9.0 |  |
| Controls, % | 8.1 |  | 9.1 |  |  | 5.2 |  | 5.7 |  |  | 3.7 |  | 4.7 |  |
| Difference (95% c.i.) | 3.1 | (2.05-4.16) | 2.1 | (1.39-2.83) |  | 5.2 | (4.78-5.57) | 3.5 | (2.53-4.57) |  | 6.0 | (3.42-4.06) | 4.3 | (2.99-5.59) |
|  |  |  |  |  |  |  |  |  |  |  |  |  |  |  |
| **Number of drugs** |  |  |  |  |  |  |  |  |  |  |  |  |  |  |
| Patients |  |  |  |  |  |  |  |  |  |  |  |  |  |  |
| mean (SD) | 7.1 | (4.1) | 6.7 | (3.7) |  | 7.2 | (4.4) | 6.5 | (3.9) |  | 5.7 | (3.7) | 6.0 |  |
| Controls |  |  |  |  |  |  |  |  |  |  |  |  |  |  |
| mean (SD) | 2.4 | (3.2) | 2.7 | (3.3) |  | 1.7 | (2.7) | 1.8 | (2.8) |  | 1.3 | (2.3) | 1.6 |  |
|  |  |  |  |  |  |  |  |  |  |  |  |  |  |  |
|  |  |  |  |  |  |  |  |  |  |  |  |  |  |  |
| **Number of outpatient visits** |  |  |  |  |  |  |  |  |  |  |  |  |  |  |
| Patients |  |  |  |  |  |  |  |  |  |  |  |  |  |  |
| mean (SD) | 5.6 | (4.0) | 2.9 | (3.9) |  | 5.7 | (4.2) | 3.1 | (3.2) |  | 5.3 | (4.3) | 3.6 |  |
| Controls |  |  |  |  |  |  |  |  |  |  |  |  |  |  |
| mean (SD) | 0.9 | (2.5) | 0.9 | (2.5) |  | 0.7 | (1.9) | 0.7 | (2.4) |  | 0.6 | (1.7) | 0.7 |  |
|  |  |  |  |  |  |  |  |  |  |  |  |  |  |  |
|  |  |  |  |  |  |  |  |  |  |  |  |  |  |  |
| **Proportion, at least one hospitalization** |  |  |  |  |  |  |  |  |  |  |  |  |  |  |
| Patients, % | 21 |  | 21 |  |  | 17 |  | 16 |  |  | 17 |  | 24 |  |
|  |  |  |  |  |  |  |  |  |  |  |  |  |  |  |
| Controls, % | 10 |  | 11 |  |  | 8 |  | 8 |  |  | 7 |  | 9 |  |

RA = Rheumatoid arthritis, PSA = psoriatic arthritis, AS = Ankylosing spondylitis, TNF = tnf-α inhibitors, NBS = Non-biological systemic drugs

Table S1b. Treatments (TNF-substance) in patients by indication for treatment

| DISEASED - TNF | Total | | RA | | PSA | | AS | |
| --- | --- | --- | --- | --- | --- | --- | --- | --- |
|  | 6,256 |  | 3,697 |  | 1,223 |  | 1,336 |  |
| Infliximab | 2,226 | 35.6% | 1,500 | 40.6% | 452 | 37.0% | 274 | 20.5% |
| Etanercept | 1,325 | 21.2% | 790 | 21.4% | 176 | 14.4% | 359 | 26.9% |
| Adalimumab | 2,118 | 33.9% | 1,007 | 27.2% | 499 | 40.8% | 612 | 45.8% |
| Certolizumab | 239 | 3.8% | 203 | 5.5% | 17 | 1.4% | 19 | 1.4% |
| Golimumab | 348 | 5.6% | 197 | 5.3% | 79 | 6.5% | 72 | 5.4% |

Table S1c. Treatments (NBS) in patients by indication for treatment

| DISEASED - NBS | Total | | RA | | PSA | | AS | |
| --- | --- | --- | --- | --- | --- | --- | --- | --- |
|  | 13,241 |  | 7,996 |  | 3,275 |  | 1,970 |  |
| Sulfasalazine | 1,493 | 11.3% | 478 | 6.0% | 446 | 13.6% | 569 | 28.9% |
| Betamethasone | 935 | 7.1% | 440 | 5.5% | 234 | 7.1% | 261 | 13.2% |
| Dexamethasone | 8 | 0.1% | 2 | 0.0% | 4 | 0.1% | 2 | 0.1% |
| Methylprednisolone | 159 | 1.2% | 79 | 1.0% | 41 | 1.3% | 39 | 2.0% |
| Prednisolone | 7,317 | 55.3% | 5,201 | 65.0% | 1,134 | 34.6% | 982 | 49.8% |
| Prednisone | 32 | 0.2% | 15 | 0.2% | 4 | 0.1% | 13 | 0.7% |
| Triamcinolone | 86 | 0.6% | 39 | 0.5% | 29 | 0.9% | 18 | 0.9% |
| Hydrocortisone | 11 | 0.1% | 3 | 0.0% | 6 | 0.2% | 2 | 0.1% |
| Cyclophosphamide | 7 | 0.1% | 3 | 0.0% | 3 | 0.1% | 1 | 0.1% |
| Methotrexate, inj | 117 | 0.9% | 63 | 0.8% | 51 | 1.6% | 3 | 0.2% |
| Mercaptopurine | 1 | 0.0% | 0 | 0.0% | 0 | 0.0% | 1 | 0.1% |
| Leflunomide | 20 | 0.2% | 13 | 0.2% | 6 | 0.2% | 1 | 0.1% |
| Ciclosporin | 14 | 0.1% | 5 | 0.1% | 8 | 0.2% | 1 | 0.1% |
| Tacrolimus | 6 | 0.0% | 4 | 0.1% | 1 | 0.0% | 1 | 0.1% |
| Azathioprine | 31 | 0.2% | 18 | 0.2% | 4 | 0.1% | 9 | 0.5% |
| Methotrexate, oral | 5,013 | 37.9% | 3,301 | 41.3% | 1,545 | 47.2% | 167 | 8.5% |
| Sodium aurothiomalate | 1 | 0.0% | 1 | 0.0% | 0 | 0.0% | 0 | 0.0% |
| Auranofin | 4 | 0.0% | 4 | 0.1% | 0 | 0.0% | 0 | 0.0% |

Sums of percentages exceed 100% since more than one substance may have been used

|  | Before start of treatment | | | | After start of treatment | | | |
| --- | --- | --- | --- | --- | --- | --- | --- | --- |
|  |  |  |  | ***reference*** |  |  |  |  |
|  | -24 months | -18 months | -12 months | -6 months | +6 months | +12 months | +18 months | +24 months |
| **N Diseased** | **6,256** | **6,256** | **6,256** | **6,256** | **6,256** | **5,685** | **4,693** | **4,027** |
| *AS* | *1,336* | *1,336* | *1,336* | *1,336* | *1,336* | *1,255* | *1,040* | *890* |
| *PS* | *1,223* | *1,223* | *1,223* | *1,223* | *1,223* | *1,129* | *938* | *800* |
| *RA* | *3,697* | *3,697* | *3,697* | *3,697* | *3,697* | *3,301* | *2,715* | *2,337* |
| **Users, ADs** | **686** | **704** | **708** | **737** | **758** | **625** | **515** | **446** |
| *AS* | *131* | *134* | *138* | *136* | *137* | *116* | *99* | *78* |
| *PS* | *169* | *169* | *172* | *184* | *201* | *159* | *134* | *108* |
| *RA* | *386* | *401* | *398* | *417* | *420* | *350* | *282* | *260* |
| **per 100** | **11.0%** | **11.3%** | **11.3%** | **11.8%** | **12.1%** | **11.0%** | **11.0%** | **11.1%** |
| *AS* | *9.8%* | *10.0%* | *10.3%* | *10.2%* | *10.3%* | *9.2%* | *9.5%* | *8.8%* |
| *PS* | *13.8%* | *13.8%* | *14.1%* | *15.0%* | *16.4%* | *14.1%* | *14.3%* | *13.5%* |
| *RA* | *10.4%* | *10.8%* | *10.8%* | *11.3%* | *11.4%* | *10.6%* | *10.4%* | *11.1%* |

Table S2. Patients starting Anti-TNF treatment, proportion with fillings of antidepressants before and after treatment start

Table S3. Controls to patients starting Anti-TNF treatment, proportion with fillings of antidepressants before and after treatment start

|  | Before start of treatment | | | | After start of treatment | | | |
| --- | --- | --- | --- | --- | --- | --- | --- | --- |
|  |  |  |  | ***reference*** |  |  |  |  |
|  | -24 months | -18 months | -12 months | -6 months | +6 months | +12 months | +18 months | +24 months |
| **N Controls** | **62,324** | **62,324** | **62,324** | **62,324** | **62,324** | **62,103** | **61,798** | **61,488** |
| *AS* | *13,282* | *13,282* | *13,282* | *13,282* | *13,282* | *13,243* | *13,184* | *13,120* |
| *PS* | *12,176* | *12,176* | *12,176* | *12,176* | *12,176* | *12,128* | *12,083* | *12,041* |
| *RA* | *36,866* | *36,866* | *36,866* | *36,866* | *36,866* | *36,732* | *36,531* | *36,327* |
| **Users, ADs** | **5401** | **5483** | **5500** | **5642** | **5,701** | **5,846** | **5,973** | **6,079** |
| *AS* | *850* | *867* | *862* | *890* | *921* | *974* | *970* | *992* |
| *PS* | *942* | *972* | *987* | *1016* | *986* | *1,029* | *1,045* | *1,084* |
| *RA* | *3609* | *3644* | *3651* | *3736* | *3,794* | *3,843* | *3,958* | *4,003* |
| **per 100** | **8.7%** | **8.8%** | **8.8%** | **9.1%** | **9.1%** | **9.4%** | **9.7%** | **9.9%** |
| *AS* | *6.4%* | *6.5%* | *6.5%* | *6.7%* | *6.9%* | *7.4%* | *7.4%* | *7.6%* |
| *PS* | *7.7%* | *8.0%* | *8.1%* | *8.3%* | *8.1%* | *8.5%* | *8.6%* | *9.0%* |
| *RA* | *9.8%* | *9.9%* | *9.9%* | *10.1%* | *10.3%* | *10.5%* | *10.8%* | *11.0%* |

Table S4. Patients starting Anti-TNF treatment, proportion with fillings of benzodiazepine related hypnotics before and after treatment start

|  | Before start of treatment | | | | After start of treatment | | | |
| --- | --- | --- | --- | --- | --- | --- | --- | --- |
|  |  |  |  | ***reference*** |  |  |  |  |
|  | -24 months | -18 months | -12 months | -6 months | +6 months | +12 months | +18 months | +24 months |
| **N Diseased** | **6,256** | **6,256** | **6,256** | **6,256** | **6,256** | **5,685** | **4,693** | **4,027** |
| *AS* | *1,336* | *1,336* | *1,336* | *1,336* | *1,336* | *1,255* | *1,040* | *890* |
| *PS* | *1,223* | *1,223* | *1,223* | *1,223* | *1,223* | *1,129* | *938* | *800* |
| *RA* | *3,697* | *3,697* | *3,697* | *3,697* | *3,697* | *3,301* | *2,715* | *2,337* |
| **Users, BRHs** | **541** | **558** | **585** | **672** | **662** | **570** | **446** | **383** |
| *AS* | *100* | *94* | *96* | *130* | *118* | *104* | *86* | *72* |
| *PS* | *106* | *118* | *116* | *127* | *133* | *120* | *101* | *83* |
| *RA* | *335* | *346* | *373* | *415* | *411* | *346* | *259* | *228* |
| **per 100** | **8.6%** | **8.9%** | **9.4%** | **10.7%** | **10.6%** | **10.0%** | **9.5%** | **9.5%** |
| *AS* | *7.5%* | *7.0%* | *7.2%* | *9.7%* | *8.8%* | *8.3%* | *8.3%* | *8.1%* |
| *PS* | *8.7%* | *9.6%* | *9.5%* | *10.4%* | *10.9%* | *10.6%* | *10.8%* | *10.4%* |
| *RA* | *9.1%* | *9.4%* | *10.1%* | *11.2%* | *11.1%* | *10.5%* | *9.5%* | *9.8%* |

Table S5. Controls to patients starting Anti-TNF treatment, proportion with fillings of benzodiazepine related hypnotics before and after treatment start

|  | Before start of treatment | | | | After start of treatment | | | |
| --- | --- | --- | --- | --- | --- | --- | --- | --- |
|  |  |  |  | ***reference*** |  |  |  |  |
|  | -24 months | -18 months | -12 months | -6 months | +6 months | +12 months | +18 months | +24 months |
| **N Controls** | **62,324** | **62,324** | **62,324** | **62,324** | **62,324** | **62,103** | **61,798** | **61,488** |
| *AS* | *13,282* | *13,282* | *13,282* | *13,282* | *13,282* | *13,243* | *13,184* | *13,120* |
| *PS* | *12,176* | *12,176* | *12,176* | *12,176* | *12,176* | *12,128* | *12,083* | *12,041* |
| *RA* | *36,866* | *36,866* | *36,866* | *36,866* | *36,866* | *36,732* | *36,531* | *36,327* |
| **Users, BRHs** | **3,768** | **3,783** | **3,968** | **4,120** | **4,202** | **4,287** | **4,328** | **4,446** |
| *AS* | *485* | *473* | *503* | *497* | *539* | *549* | *558* | *555* |
| *PS* | *589* | *598* | *621* | *630* | *659* | *681* | *678* | *708* |
| *RA* | *2,694* | *2,712* | *2,844* | *2,993* | *3,004* | *3,057* | *3,092* | *3,183* |
| **per 100** | **6.0%** | **6.1%** | **6.4%** | **6.6%** | **6.7%** | **6.9%** | **7.0%** | **7.2%** |
| *AS* | *3.7%* | *3.6%* | *3.8%* | *3.7%* | *4.1%* | *4.1%* | *4.2%* | *4.2%* |
| *PS* | *4.8%* | *4.9%* | *5.1%* | *5.2%* | *5.4%* | *5.6%* | *5.6%* | *5.9%* |
| *RA* | *7.3%* | *7.4%* | *7.7%* | *8.1%* | *8.1%* | *8.3%* | *8.5%* | *8.8%* |

Table S6. Patients starting non-biological systemics, proportion with fillings of antidepressants (ADs) before and after treatment start

|  | Before start of treatment | | | | After start of treatment | | | |
| --- | --- | --- | --- | --- | --- | --- | --- | --- |
|  |  |  |  | ***reference*** |  |  |  |  |
|  | -24 months | -18 months | -12 months | -6 months | +6 months | +12 months | +18 months | +24 months |
| **N Diseased** | **13,241** | **13,241** | **13,241** | **13,241** | **13,241** | **10,026** | **8,076** | **6,681** |
| *AS* | *1,970* | *1,970* | *1,970* | *1,970* | *1,970* | *1,012* | *680* | *481* |
| *PS* | *3,275* | *3,275* | *3,275* | *3,275* | *3,275* | *2,330* | *1,800* | *1,464* |
| *RA* | *7,996* | *7,996* | *7,996* | *7,996* | *7,996* | *6,684* | *5,596* | *4,736* |
| **Users, ADs** | **1,302** | **1,340** | **1,385** | **1,449** | **1,405** | **1,006** | **841** | **692** |
| *AS* | *202* | *203* | *213* | *230* | *212* | *106* | *75* | *58* |
| *PS* | *366* | *372* | *381* | *403* | *399* | *256* | *199* | *159* |
| *RA* | *734* | *765* | *791* | *816* | *794* | *644* | *567* | *475* |
| **per 100** | **9.8%** | **10.1%** | **10.5%** | **10.9%** | **10.6%** | **10.0%** | **10.4%** | **10.4%** |
| *AS* | *10.3%* | *10.3%* | *10.8%* | *11.7%* | *10.8%* | *10.5%* | *11.0%* | *12.1%* |
| *PS* | *11.2%* | *11.4%* | *11.6%* | *12.3%* | *12.2%* | *11.0%* | *11.1%* | *10.9%* |
| *RA* | *9.2%* | *9.6%* | *9.9%* | *10.2%* | *9.9%* | *9.6%* | *10.1%* | *10.0%* |

Table S7. Controls to patients starting non-biological systemics, proportion with fillings of antidepressants before and after treatment start

|  | Before start of treatment | | | | After start of treatment | | | |
| --- | --- | --- | --- | --- | --- | --- | --- | --- |
|  |  |  |  | ***reference*** |  |  |  |  |
|  | -24 months | -18 months | -12 months | -6 months | +6 months | +12 months | +18 months | +24 months |
| **N Controls** | **132,113** | **132,113** | **132,113** | **132,113** | **132,113** | **131,794** | **131,064** | **130,173** |
| *AS* | *19,629* | *19,629* | *19,629* | *19,629* | *19,629* | *19,574* | *19,484* | *19,484* |
| *PS* | *32,654* | *32,654* | *32,654* | *32,654* | *32,654* | *32,596* | *32,476* | *32,476* |
| *RA* | *79,830* | *79,830* | *79,830* | *79,830* | *79,830* | *79,624* | *79,104* | *79,104* |
| **Users, ADs** | **11,703** | **11,890** | **12,102** | **12,333** | **12,649** | **12,894** | **13,164** | **13,408** |
| *AS* | *1,347* | *1,366* | *1,413* | *1,402* | *1,486* | *1,480* | *1,526* | *1,530* |
| *PS* | *2,570* | *2,585* | *2,608* | *2,670* | *2,720* | *2,752* | *2,814* | *2,921* |
| *RA* | *7,786* | *7,939* | *8,081* | *8,261* | *8,443* | *8,662* | *8,824* | *8,957* |
| **per 100** | **7.0%** | **7.2%** | **7.4%** | **7.6%** | **7.9%** | **8.1%** | **8.2%** | **8.5%** |
| *AS* | *6.9%* | *7.0%* | *7.2%* | *7.1%* | *7.6%* | *7.6%* | *7.8%* | *7.9%* |
| *PS* | *7.9%* | *7.9%* | *8.0%* | *8.2%* | *8.3%* | *8.4%* | *8.7%* | *9.0%* |
| *RA* | *9.8%* | *9.9%* | *10.1%* | *10.3%* | *10.6%* | *10.9%* | *11.2%* | *11.3%* |

Table S8. Patients starting non-biologica systemics, proportion with fillings of benzodiazepine related hypnotics (BRH) before and after treatment start

|  | Before start of treatment | | | | After start of treatment | | | |
| --- | --- | --- | --- | --- | --- | --- | --- | --- |
|  |  |  |  | ***reference*** |  |  |  |  |
|  | -24 months | -18 months | -12 months | -6 months | +6 months | +12 months | +18 months | +24 months |
| **N Diseased** | **13,241** | **13,241** | **13,241** | **13,241** | **13,241** | **10,026** | **8,076** | **6,681** |
| *AS* | *1,970* | *1,970* | *1,970* | *1,970* | *1,970* | *1,012* | *680* | *481* |
| *PS* | *3,275* | *3,275* | *3,275* | *3,275* | *3,275* | *2,330* | *1,800* | *1,464* |
| *RA* | *7,996* | *7,996* | *7,996* | *7,996* | *7,996* | *6,684* | *5,596* | *4,736* |
| **Users, BRHs** | **1,057** | **1,132** | **1,209** | **1,378** | **1,503** | **1,033** | **796** | **677** |
| *AS* | *140* | *157* | *158* | *178* | *196* | *94* | *62* | *43* |
| *PS* | *241* | *250* | *279* | *302* | *340* | *211* | *155* | *116* |
| *RA* | *676* | *725* | *772* | *898* | *967* | *728* | *579* | *518* |
| **per 100** | **8.0%** | **8.5%** | **9.1%** | **10.4%** | **11.4%** | **10.3%** | **9.9%** | **10.1%** |
| *AS* | *7.1%* | *8.0%* | *8.0%* | *9.0%* | *9.9%* | *9.3%* | *9.1%* | *8.9%* |
| *PS* | *7.4%* | *7.6%* | *8.5%* | *9.2%* | *10.4%* | *9.1%* | *8.6%* | *7.9%* |
| *RA* | *8.5%* | *9.1%* | *9.7%* | *11.2%* | *12.1%* | *10.9%* | *10.3%* | *10.9%* |

Table S9. Controls to patients starting non-biological systemics, proportion with fillings of benzodiazepine related hypnotics (BRH) before and after treatment start

|  | Before start of treatment | | | | After start of treatment | | | |
| --- | --- | --- | --- | --- | --- | --- | --- | --- |
|  |  |  |  | ***reference*** |  |  |  |  |
|  | -24 months | -18 months | -12 months | -6 months | +6 months | +12 months | +18 months | +24 months |
| **N Controls** | **132,113** | **132,113** | **132,113** | **132,113** | **132,113** | **131,794** | **131,064** | **130,173** |
| *AS* | *19,629* | *19,629* | *19,629* | *19,629* | *19,629* | *19,574* | *19,484* | *19,484* |
| *PS* | *32,654* | *32,654* | *32,654* | *32,654* | *32,654* | *32,596* | *32,476* | *32,476* |
| *RA* | *79,830* | *79,830* | *79,830* | *79,830* | *79,830* | *79,624* | *79,104* | *79,104* |
| **Users, BRHs** | **9,197** | **9,485** | **9,812** | **10,068** | **10,421** | **10,711** | **10,794** | **11,008** |
| *AS* | *1,347* | *1,366* | *1,413* | *1,402* | *1,486* | *1,480* | *1,526* | *1,530* |
| *PS* | *2,570* | *2,585* | *2,608* | *2,670* | *2,720* | *2,752* | *2,814* | *2,921* |
| *RA* | *7,786* | *7,939* | *8,081* | *8,261* | *8,443* | *8,662* | *8,824* | *8,957* |
| **per 100** | **7.0%** | **7.2%** | **7.4%** | **7.6%** | **7.9%** | **8.1%** | **8.2%** | **8.5%** |
| *AS* | *6.9%* | *7.0%* | *7.2%* | *7.1%* | *7.6%* | *7.6%* | *7.8%* | *7.9%* |
| *PS* | *7.9%* | *7.9%* | *8.0%* | *8.2%* | *8.3%* | *8.4%* | *8.7%* | *9.0%* |
| *RA* | *9.8%* | *9.9%* | *10.1%* | *10.3%* | *10.6%* | *10.9%* | *11.2%* | *11.3%* |
